# Untapped Potential for Emergency Department Observation Unit Use: A National Hospital Ambulatory Medical Care Survey (NHAMCS) Study

**DOI:** 10.5811/westjem.2021.8.52231

**Published:** 2022-01-18

**Authors:** Angelo Navas, Billy Guzman, Almujtaba Hassan, Joseph B. Borawski, Dean Harrison, Pratik Manandhar, Alaatin Erkanli, Alexander T. Limkakeng

**Affiliations:** * Duke University School of Medicine, Department of Emergency Medicine, Durham, North Carolina; † King Fahad Armed Forces Hospital, Department of Critical Care Medicine, Jeddah, Kingdom of Saudi Arabia; ‡ Duke University Medical Center, Duke Clinical Research Institute, Durham, North Carolina; § Duke University School of Medicine, Department of Biostatistics and Bioinformatics, Durham, North Carolina

## Abstract

**Introduction:**

Millions of people present to the emergency department (ED) with chest pain annually. Accurate and timely risk stratification is important to identify potentially life-threatening conditions such as acute coronary syndrome (ACS). An ED-based observation unit can be used to rapidly evaluate patients and reduce ED crowding, but the practice is not universal. We estimated the number of current hospital admissions in the United States (US) eligible for ED-based observation services for patients with symptoms of ACS.

**Methods:**

In this cross-sectional analysis we used data from the 2011–2015 National Hospital Ambulatory Medical Care Survey (NHAMCS). Visits were included if patients presented with symptoms of ACS (eg, chest pain, dyspnea), had an electrocardiogram (ECG) and cardiac markers, and were admitted to the hospital. We excluded patients with any of the following: discharge diagnosis of myocardial infarction; cardiac arrest; congestive heart failure, or unstable angina; admission to an intensive care unit; hospital length of stay > 2 days; alteplase administration, central venous catheter insertion, cardiopulmonary resuscitation or endotracheal intubation; or admission after an initial ED observation stay. We extracted data on sociodemographics, hospital characteristics, triage level, disposition from the ED, and year of ED extracted from the NHAMCS. Descriptive statistics were performed using sampling weights to produce national estimates of ED visits. We provide medians with interquartile ranges for continuous variables and percentages with 95% confidence intervals for categorical variables.

**Results:**

During 2011–2015 there were an estimated 675,883,000 ED visits in the US. Of these, 14,353,000 patients with symptoms of ACS and an ED order for an ECG or cardiac markers were admitted to the hospital. We identified 1,883,000 visits that were amenable to ED observation services, where 987,000 (52.4%) were male patients, and 1,318,000 (70%) were White. Further-more, 739,000 (39.2%) and 234,000 (12.4%) were paid for by Medicare and Medicaid, respectively. The majority (45.1%) of observation-amenable hospitalizations were in the Southern US.

**Conclusion:**

Emergency department-based observation unit services for suspected ACS appear to be underused. Over half of potentially observation-amenable admissions were paid for by Medicare and Medicaid. Implementation of ED-based observation units would especially benefit hospitals and patients in the American South.

## INTRODUCTION

Over six million adults present to the emergency department (ED) with chest pain in the United States annually.^1^,^2^ While there are multiple etiologies of chest pain, including non-cardiac and benign disorders, accurate and timely risk stratification is important to identify potentially life-threatening conditions such as acute coronary syndrome (ACS). Several objective measures (ie, electrocardiography [ECG], cardiac biomarkers, noninvasive imaging of the myocardium)^3^ and decision-support tools^4^ have been developed for ACS risk stratification. Yet 2–4% of patients with ACS are inadvertently discharged from the ED.^5^,^6^,^7^ Of those patients with chest pain admitted for further evaluation, less than half will be diagnosed with ACS.^8^ One factor contributing to these discrepancies in the ED is that ACS symptoms are often non-specific.^9^ Additionally, multiple non-ACS conditions are associated with elevated troponin levels.^10^ Emerging evidence suggests that ED-based observation units (EDOU) for chest pain may overcome these limitations by enabling implement-ation of a rapid risk-stratification protocol (eg, cardiac biomarker testing, telemetry monitoring, stress testing, echocardiogram) over a short period of time.^1^,^11^

The use of EDOUs has been described since the 1980s. In 2006 an Institute of Medicine report, *The Future of Emergency Care in the United States Health System*, supported the use of EDOUs as a tool to reduce ED crowding, improve patient care, and reduce cost.^12^ Although these units are diverse, a defining feature is the use of protocolized care with the goal of rapidly discharging the patient back home within 24 hours. Despite documented financial and patient benefits, their adoption has not been universal.^13^ Recent estimates suggest that 39% of EDs have a separate observation or clinical decision unit.^2^ While the utility of EDOUs for chest pain has been reported,^4^,^14^ it is not fully known to what degree ED-based observation services could expand in the United States.

In this study we used a publicly available, de-identified, and unlinked survey database of nationwide ED visits to determine the number of patients admitted to the hospital from the ED for symptoms of ACS who could potentially have been evaluated in an EDOU. We also sought to determine which patient factors were most associated with patients being admitted despite meeting our derived observation-eligible criteria. Our goal was to corroborate the potential for more EDOUs nationwide as a means to significantly reduce unnecessary hospital admissions and related expenses.

## METHODS

### Study Design and Data Source

We conducted a cross-sectional analysis using data from the National Hospital Ambulatory Medical Care Survey (NHAMCS) from 2011–2015. Visits were included if patients presented with symptoms of ACS (eg, chest pain, dyspnea), had an electrocardiogram (ECG) and cardiac markers, and were admitted to the hospital. We obtained data for this analysis from the publicly available NHAMCS dataset published on the US Centers for Disease Control and Prevention (CDC) website. The database includes information that is de-identified and unlinked to the patient encounter and aggregated solely for informative and research purposes.

**Population Health Research Capsule**

What do we already know about this issue?

*Delay in rapid identification of potentially life-threatening conditions such as acute coronary syndrome (ACS) can be secondary to ED crowding.*

What was the research question?

*What would be the benefit of ED-based observation units (EDOU) for suspected ACS?*

What was the major finding of the study?

*Implementaion of EDOUs would benefit patients with suspected ACS, especially in the Southern US.*

How does this improve population health?

*An EDOU could minimize ED crowding and rapidly identify potentially life-threatening conditions such as ACS, and could have economic impact nationwide.*

The NHAMCS is an annual, nationally representative probability sample survey administered by the CDC’s National Center for Health Statistics. Data is collected on visits to outpatient clinics and EDs of non-institutional, short-stay. and general hospitals in 50 states and the District of Columbia, excluding federal, military, and Veterans Affairs hospitals. The NHAMCS uses a four-stage probability sampling design including selection of primary sampling units (PSU), hospitals within PSUs, clinics within hospitals, and patient visits within clinics. The exact methods of the NHAMCS survey have been described in detail elsewhere.^15^

Hospitals are selected based on geographic PSUs. For the years included, on average 411 hospitals were eligible annually, and 369 participated, giving an unweighted average hospital sampling response rate of 89.8%. Sixteen data collection groups randomly rotate across these hospitals through 13 four-week reporting periods throughout the year. Contractors for the NHAMCS (SRA International, Inc., Durham, NC) collect data from ED visit medical records while being monitored by NHAMCS field representatives. The NHAMCS staff members independently check 10% of the data for accuracy. Error rates are 0.3–0.9% for various items on the survey; the survey includes patient-level data, patient disposition, and hospital-level data.

### Study Population

**Figure 1:**
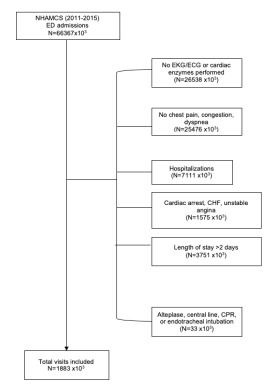
Flow diagram of inclusion and exclusion criteria for patient visits associated with acute coronary syndrome care. *NHAMCS*, National Hospital Ambulatory Medical Care Survey; *ED*, emergency department; *EKG/ECG*, electrocardiogram; *CHF*, congestive heart failure; *CPR*, cardiopulmonary resuscitation.

For this analysis we focused on visits to hospital EDs for symptoms of ACS (Figure 1). Visits were included if a cardiac troponin and ECG were ordered in the ED, the patient was admitted to inpatient status into the hospital and was discharged with a length of stay shorter than two days. We excluded patients with any of the following: discharge diagnosis of myocardial infarction, cardiac arrest, congestive heart failure, or unstable angina; admission to an intensive care unit; hospital length of stay > 2 days; alteplase administration, central venous catheter insertion, cardiopulmonary resuscitation or endotracheal intubation; or admission after an initial ED observation stay. We further excluded any patient with a non-cardiovascular primary diagnosis upon hospital discharge.

### Variables

The NHAMCS survey records demographic data, payment source, clinician types, procedures, prescriptions, laboratory and radiographic tests ordered for each visit, up to three reasons for visit (chief complaints), the ED diagnosis (*International Classification of Diseases, 9^th^ Revision* codes), and the final hospital discharge diagnosis for those patients who were admitted to the hospital. In addition, we extracted the following variables from the NHAMCS database: age; race/ethnicity; gender; insurance status; clinician type; hospital characteristics (geographic location at the level of state, academic status, metropolitan area, and ownership); and disposition from the ED (admission, discharge, and transfer). We used data from the NHAMCS files for 2011–2015.

### Ethics

This study was exempted from full review by our institutional review board.

### Data Analysis

We performed data analyses using SAS version 9.4 (SAS Institute Inc., Cary, NC). Sociodemographics, hospital characteristics, and dispositions from the ED were summarized using the median with interquartile range for continuous variables and percentage with 95% confidence intervals for categorical variables. We calculated unweighted percentages for “reasons for visits” and final diagnoses. Estimates of average annual national visits were derived using survey procedures with the weights, strata, and PSU design variables provided by the NHAMCS.

## RESULTS

**Figure 2:**
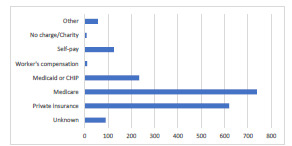
Source of payment for emergency department visits amenable to observation services.

**Figure 3:**
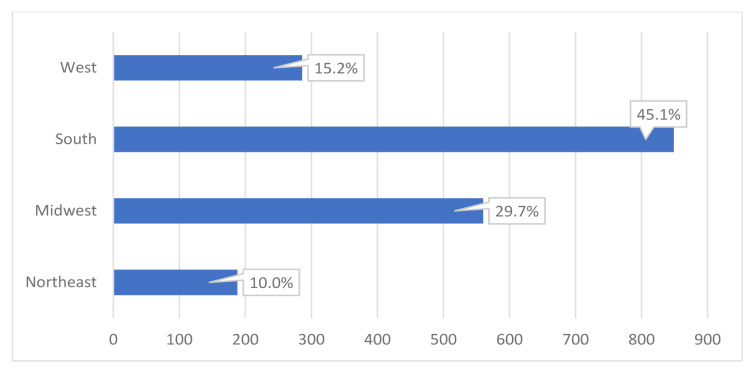
Geographic region of emergency department visits amenable to observation services.

During 2011–2015, there were an estimated 675,883,000 ED visits nationwide in the US. Of these, 14,353,000 patients with symptoms of ACS who had an ECG in the ED and cardiac markers were admitted to the hospital. This number was calculated using raw percentages of selective cohorts and population ratio adjustment. We identified 1,883,000 visits that may have been amenable to observation services. Of these visits, 987,000 (52.4 %) were by males and 1,318,000 (70.0 %) identified as White (Table 1). Furthermore, 739,000 (39.2 %) and 234,000 (12.4 %) were paid for by Medicare and Medicaid, respectively (Figure 2). The majority of these observation-amenable hospitalizations were in the Southern US (Figure 3).

When comparing ED visits leading to observation-amenable admissions to overall proportions of ED visits, they occurred proportionally slightly more in females, Medicare patients, and in the Midwest and South. These types of admissions occurred less often in Medicaid patients and in the US Northeast and West.

## DISCUSSION

Chest pain is the second most common reason for ED visits in the US. An EDOU can be particularly useful to risk-stratify patients with symptoms of ACS. These units provide a period of therapeutic intervention and diagnostics (usually 24 hours) as an alternative to hospitalization where the appropriateness of inpatient services is unclear. The EDOU protocols can reduce healthcare costs and help vulnerable patients with common cardiac complaints avoid unnecessary hospitalizations.^9^,^12^,^16^ However, there is no clear estimate of annual ED visits in the US for ACS symptoms that would be amenable to evaluation in the EDOU or the characteristics of such visits. The current analysis provides an estimate of the need for systematic national efforts to encourage the implementation of EDOUs to evaluate patients with ACS symptoms.

In this study we determined the proportion of patients who were hospitalized for symptoms of potential ACS who could have been observed in an EDOU. Emergency department-based observation unit services for ACS appear to be underused. We identified that over half of observation-amenable admissions were paid for by Medicare and Medicaid. These findings appear to vary geographically within the US.

This is the first study based on a nationally representative sample to evaluate the rate of use of EDOUs for chest pain. Previous work has shown that national EDOUs are growing in number, from 19% in 2003 to 39% in 2017, and that chest pain is the most common EDOU symptom requiring diagnosis.^2^,^14^,^16^ Although the number of EDOUs is growing, there remains room for further expansion. Studies have shown that patients at low risk or intermediate risk of ACS are more common than patients with unstable angina or ST-elevation myocardial infarction.^17^ Indeed, we identified nearly two million patients within this risk category who were not evaluated in an EDOU. Importantly, EDOUs have been shown to reduce admission rates from the ED for chest pain.^18^ Another benefit of the EDOU is reduction in costs, with an estimated savings of $124 per patient for ED visits with chest pain.^19^

The majority of patients in our analysis who missed an opportunity for EDOU evaluation were Medicare beneficiaries. Older patients (age >65 years) have a higher rate of EDOU use for chest pain.^20^ Previous work has specifically determined how much Medicaid and Medicare have paid for observation services.^21^ These studies show that patients treated in observation units for chest pain are less likely to have an adverse event within 30 days and Medicare payments for these services are nearly half what they are for inpatient services.^22^ On the other hand, these services may cost patients more because they fall under Medicare Part B (outpatient-related services), which requires them to pay a $183 deductible and 20% cost-sharing for services.^22^ We also note that observation-amenable admissions occur proportionally more frequently in Medicare patients and less frequently in Medicaid patients, suggesting that financial incentives may play a role. Thus, it is possible that although EDOUs reduce a hospital’s health-related expenditures, one reason for their underutilization is patient finances. It is important to note, however, that for commercially insured patients, observation units reduce total out-of-pocket expenses.^23^ Increasing the use of observation services may require further expanding the number of EDOUs in the US and evaluating payment options for Medicare beneficiaries.

Despite the growing use of EDOUs, no previous work has specifically commented on geographic variation of observation for ACS.^24^ However, studies have shown over half of EDOUs generally are in an urban setting and 30% are in the South.^25^ Our analysis revealed that 45% of the visits for ACS that could have been seen in an EDOU were in hospitals in the Southeast US, while just 10% were in the Northeast US. One explanation for these differences may be that there are a greater number of EDOUs or a shorter distance to a hospital with an ED in the Northeast – an important factor when a patient is experiencing symptoms of ACS. It is also possible that many of the EDs in the Northeast and West are in high-density population areas and have already adapted EDOUs as a means of dealing with hospital crowding. An investigation of ED-managed observation units also found that EDs in an area with a median income of <$32,000 were less likely to have an observation unit.^26^ This is particularly important given research showing that patients with a lower socioeconomic status may report to EDs less frequently for chest pain or delay seeking treatment for chest pain.^26^,^27^

## LIMITATIONS

Our study has several limitations. First, this was a cross-sectional analysis using retrospective data of ED visits across five years (2011–2015). However, the NHAMCS is a nationally representative database that includes data on patient visits to the ED, demographic characteristics, symptoms, chief complaint, diagnoses, laboratory services, and medications. We recognize that we present a hypothetical construct of what an observation-amenable admission is, but we believe it is a reasonable estimate. This database estimate cannot account for numerous factors that may have impacted needs for hospitalization and were not in the existing NHAMCS variables, including social determinants of health. Furthermore, it represents a conservative estimate of the number of hospitalizations potentially amenable to observation services.

We did not include patients hospitalized for other, atypical anginal symptoms, and it may have been clinically appropriate and reasonable for a certain proportion of the hospitalizations that we excluded to have started out as observation status. Next, the unweighted ED response rate of the NHAMCS was <80%, which could have biased results and limited generalizability. Nevertheless, the NHAMCS is the largest dataset to date with population-based estimates of ED visits in the US. Finally, while our inclusion criteria may be subject to misclassification bias, we used a comprehensive algorithm to determine participants with ACS symptoms who were not evaluated in an EDOU. It is also important to note that not all hospitals can build and staff observation units easily.

## CONCLUSION

We identified 1,883,000 visits for ACS symptoms that were amenable to EDOU services. We also found that over half of observation-amenable admissions were paid for by Medicare and Medicaid, and were more likely to occur in the Southern US. These data support the need to further expand the use of EDOU for patients with symptoms of ACS. Although the benefits of ED-based observation service have been previously modeled theoretically and demonstrated in local settings, ultimately, further research should determine the economic and patient-oriented impacts of expansion of ED-based observation services as it actually occurs nationwide.

## Figures and Tables

**Table 1:** Characteristics of emergency department visits amenable to observation services.

Characteristic	Weighted number (x10^3^)	Weighted proportion of admissions potentially amenable to observation, % (95% CI)	Weighted number in all ED visits (x10^3^)	Weighted proportion in all ED visits (%)
Age				
Median	1883	56.4 (53.9, 58.8)	675,883	100%
25th		47.0 (43.9, 50.2)		
75th		68.5 (64.1, 72.9)		
Race/ethnicity				
Non-Hispanic White	1318	70.0 (63.6, 76.3)	396,617	58.7%
Non-Hispanic Black	369	19.6 (13.9, 25.3)	153,018	22.6%
Hispanic	149	7.9 (4.8, 11.0)	105,988	15.7%
Non-Hispanic Other	47	2.5 (0.1, 5.0)	20,260	3.0%
Gender				
Female	896	47.6 (41.3, 53.9)	373,717	55.3%
Male	987	52.4 (46.1, 58.7)	302,165	44.7%
Unknown	90	4.8 (1.7, 7.8)		
Primary source of payment				
Private insurance	620	32.9 (26.9, 39.0)	190,986	28.3%
Medicare	739	39.2 (32.8, 45.7)	123,652	18.3%
Medicaid or CHIP	234	12.4 (8.4, 16.5)	192,110	28.4%
Workers’ compensation	10	0.6 (0.0, 1.6)	55,35	0.8%
Self-pay	125	6.6 (3.7, 9.5)	85,766	12.7%
No charge/Charity	8	0.4 (0.0, 0.9)	5,721	0.8%
Other	57	3.0 (0.4, 5.6)	18,907	2.8%
Seen in this ED within last 72 hours				
Unknown	137	7.2 (2.9, 11.6)	70,984	10.5%
Yes	33	1.8 (0.5, 3.0)	28,233	4.2%
No	1714	91.0 (86.5, 95.5)	567,103	83.9%
Geographic region				
Northeast	188	10.0 (6.8, 13.1)	116,551	17.2%
Midwest	560	29.7 (22.2, 37.3)	159,356	23.6%
South	849	45.1 (36.7, 53.5)	257,543	38.1%
West	286	15.2 (10.9, 19.5)	142,432	21.1%

*ED*, emergency department; *CI*, confidence interval; *CHIP*, Children’s Health Insurance Program.

## References

[1] Shah J, Shah A, Pietrobon R (2009). Scientific writing of novice researchers: what difficulties and encouragements do they encounter?. Acad Med.

[2] Rui P, Kang K (2017). National Hospital Ambulatory Medical Care Survey: 2017 emergency department summary tables.

[3] Mokhtari A, Dryver E, Soderholm M (2015). Diagnostic values of chest pain history, ECG, troponin and clinical gestalt in patient with chest pain and potential acute coronary syndrome assesses in the emergency department. Springerplus.

[4] Gesell SB, Golden SL, Limkaken AT (2018). Implementation of the HEART Pathway: using the consolidated framework for implementation research. Crit Pathw Cardiol.

[5] Pope JH, Aufderheide TP, Ruthazer R (2000). Missed diagnoses of acute cardiac ischemia in the emergency department. N Engl J Med.

[6] Schull MJ, Vermeulen MJ, Stukel TA (2006). The risk of missed diagnosis of acute myocardial infarction associated with Emergency Department volume. Ann Emerg Med.

[7] Lee TH, Rouan GW, Weisberg MC (1987). Clinical characteristics and natural history of patients with acute myocardial infarction sent home from the emergency room. Am J Cardiol.

[8] Hansen LH, Mikkelsen S (2013). Ischemic heart disease: accuracy of the prehospital diagnosis – a retrospective study. Emerg Med Int.

[9] Borawski JB, Graff L, Limkaken AT (2017). Care of the patient with chest pain in the observation unit. Emerg Med Clin North Am.

[10] Januzzi JL (2010). Causes of non ACS related troponin elevations.

[11] Amsterdam EA, Wenger NK, Brindis RG (2014). 2014 AHA/ACC Guideline for the management of patients with non-ST-elevation acute coronary syndromes: Executive summary. Circulation.

[12] Berwanger O, Polanczyk CA, Rosito G (2017). Chest pain observation units for patients with symptoms suggestive of acute cardiac ischemia. Cochrane Database Sys Rev.

[13] Institute of Medicine (2006). The future of emergency care in the United States health system. Ann Emerg Med.

[14] Ross MA, Aurora T, Graff L (2012). State of the art: emergency department observation units. Crit Pathw Cardiol.

[15] Madsen T, Mallin M, Bledsoe J (2009). Utility of the emergency department observation unit in ensuring stress testing in low-risk chest pain patients. Crit Pathw Cardiol.

[16] McNaughton CD, Self WH (2013). Observational health services studies using nationwide administrative datasets: understanding strengths and limitations of the National Hospital Ambulatory Medical Care Survey (NHAMCS). Ann Emerg Med.

[17] Kirk JD, Diercks DB, Turnipseed SD (2000). Evaluation of chest pain suspicious for acute coronary syndrome: use of an accelerated diagnostic protocol in a chest pain evaluation unit. Am J Cardiol.

[18] Goodacre S, Nicholl J, Dixon S (2004). Randomised controlled trial and economic evaluation of a chest pain observation unit compared with routine care. BMJ.

[19] Graff L, Delara J, Ross M Chest Pain Evaluation Registry (CHEPER) Study: impact on the care of the emergency department chest pain patient. Am J Cardiol.

[20] Ross MA, Compton S, Richardson D (2003). The use and effectiveness of an emergency department observation unit for elderly patients. Ann Emerg Med.

[21] Cafardi SG, Pines JM, Deb P (2016). Increased observation services in Medicare beneficiaries with chest pain. Am J Emerg Med.

[22] Centers for Medicare and Medicaid Services (2014). Are you a hospital inpatient or outpatient?.

[23] Adrion ER, Kocher KE, Nallamothu BK (2017). Rising use of observation care among the commercially insured may lead to total and out-of-pocket cost savings. Health Affairs.

[24] Napolitano JD, Saini I (2014). Observation Units: Definition, History, Data, Financial Considerations, and Metrics. Curr Emerg Hosp Med Rep 2.

[25] Wiler JL, Ross MA, Ginde AA (2011). National study of emergency department observation services. Acad Emerg Med.

[26] Richards HM, Reid ME, Murray Watt GC (2002). Socioeconomic variations in responses to chest pain: qualitative study. BMJ.

[27] Wechkunanukul K, Grantham H, Damarell R (2016). The association between ethnicity and delay in seeking medical care for chest pain: a systematic review. JBI Database System Rev Implement Rep.

